# The mitochondrial genome of *Endoconidiophora resinifera* is intron rich

**DOI:** 10.1038/s41598-018-35926-y

**Published:** 2018-12-04

**Authors:** Abdullah Zubaer, Alvan Wai, Georg Hausner

**Affiliations:** Department of Microbiology, University of Manitoba, Winnipeg, MB R3T 2N2 Canada

## Abstract

*Endoconidiophora resinifera* (=*Ceratocystis resinifera*) is a blue-stain fungus that occurs on conifers. The data showed that the *Endoconidiophora resinifera* mitochondrial genome is one of the largest mitochondrial genomes (>220 kb) so far reported among members of the Ascomycota. An exceptional large number of introns (81) were noted and differences among the four strains were restricted to minor variations in intron numbers and a few indels and single nucleotide polymorphisms. The major differences among the four strains examined are due to size polymorphisms generated by the absence or presence of mitochondrial introns. Also, these mitochondrial genomes encode the largest cytochrome oxidase subunit 1 gene (47.5 kb) reported so far among the fungi. The large size for this gene again can be attributed to the large number of intron insertions. This study reports the first mitochondrial genome for the genus *Endoconidiophora*, previously members of this genus were assigned to *Ceratocystis*. The latter genus has recently undergone extensive taxonomic revisions and the mitochondrial genome might provide loci that could be applied as molecular markers assisting in the identification of taxa within this group of economically important fungi. The large mitochondrial genome also may provide some insight on mechanisms that can lead to mitochondrial genome expansion.

## Introduction

*Endoconidiophora resinifera* (=*Ceratocystis resinifera*) is a fungus that belongs to the Ceratocystidaceae (Sordariomycetes, Microascales). It is associated with causing blue-stain on sapwood that ultimately leads to the discolouration of timber and timber-derived products. Blue-stain is considered one of the major causes of loss in value of conifer timber as it restricts its export potential and discoloured timber is less desirable for high end use^1^. Some members of the genus *Ceratocystis sensu lato* (recently subdivided into several new genera including *Endoconidiophora*^2^) are known for causing infections such as black rot disease in sweet potato (*Ceratocystis fimbriata*)^3^, oak wilt (*Ceratocystis fagacearum*)^4^, wilt in cacao plant (*Ceratocystis cacaofunesta*)^5^, canker stain of plane trees (*Ceratocystis platani*)^6^, and sapstreak in maple tree (*Ceratocystis virescens*)^7^. *Endoconidiofora resinifera* has not been associated with any pathogenicity but this insect vectored fungus can colonize bark beetle galleries and wounds in species of *Picea*^8^. Species of *Ceratocystis s*.*l*. have been studied with regards to their taxonomy, blue-staining ability, and pathology^9^, but so far only *C*. *cacaofunesta* has been examined in more detail with regards to genomic investigations^10^. Previous studies on this group of fungi with regards to mitochondrial DNA focused on the rRNA genes and these displayed a large variety of intron insertions among various *Ceratocystis s*.*l*. species^11,12^. Additional mitochondrial genomes have recently been sequenced for members of *Ceratocystis* but so far a detailed annotation is only available for the mitochondrial genome of *C*. *cacaofunesta*^10,13–16^.

Fungal mitochondrial genomes usually encode genes involved in translation [small and large ribosomal subunit RNAs (*rns* and *rnl*) and tRNAs], proteins involved in the respiratory chain [subunits for Complex III and Complex IV (*cob*, *cox1*, *cox2*, and *cox3*)], subunits of NADH dehydrogenase (*nad1* to *nad6* and *nad4L*; except for members of the Taphrinomycota and some members of the Saccharomycetales), plus some of the components of the ATP synthase (*atp6*, *atp8*, and usually *atp9*), and in some instances the ribosomal protein RPS3^17,18^. Mitochondrial genome sizes among the fungi are quite variable ranging from 12.055 kb (in *Rozella allomycis*^19^) to 235 kb (in *Rhizoctonia solani*^20^). Mitochondrial genome size variation has also been reported among closely related species^21^. The size variations are mainly due to the number and sizes of intron insertions and size of intergenic spacers. Gene order, repeats, and in some instances other types of elements such as plasmid insertions are additional sources that generate variability among fungal mitochondrial genomes^22^.

Fungal mitochondrial introns, based on structure and splicing mechanisms, can be assigned to either group I or group II introns. These elements are potential ribozymes that can in part catalyze their own removal from transcripts; in addition these introns can encode open reading frames (ORFs) for so-called intron-encoded proteins (IEPs). Fungal mitochondrial group I introns tend to encode GIY-YIG or LAGLIDADG homing endonuclease genes (HEGs), and group II introns typically encode reverse transcriptase (RT) genes^23^. These IEPs tend to catalyze the mobility of their respective introns from an intron-plus to an intron-minus cognate allele. Some IEPs have been shown to assist in the splicing of the introns that encode them by providing so called maturase activity; i.e. these IEPs promote the folding of the intron RNA into a splicing competent structure^24,25^. Various nuclear genome-encoded factors also have been co-opted to assist in the splicing of mitochondrial introns^26^.

Group I introns primarily mobilize via a DNA-based mechanism that involves its IEP [homing endonucleases (HEases)] generating a double-stranded cut at a cognate allele that is repaired by the double-strand break repair system. This involves homologous recombination using the intron-plus allele as the repair template and in a nonreciprocal manner the intron sequence and sometimes some flanking markers are transferred to repair the double-stranded break. Group II introns primarily act like retroelements where the mobility pathway utilizes an RNA intermediate and reverse transcriptase activity. In general, mobility of these group I and group II introns is referred to as homing or retrohoming, respectively, as they tend to invade cognate alleles that have not yet been invaded at a particular site. However, these elements can potentially insert into new locations (ectopic integration) here the terms transposition or retro-transposition (for group II introns) are applicable^17^. Mobile introns and homing endonuclease genes are sometimes referred to as selfish DNAs or selfish genes as they do not appear to benefit the genomes that host these elements. It is generally assumed that mobile introns are neutral with regards to phenotype thus ensuring their survival^26^. However, being “neutral” (i.e. evolving by drift), can result in the rapid degeneration of these elements due to a lack of selection. In order for long term persistence, these elements have to invade intron-less alleles or invade new sites^27^. There is also considerable evidence that these elements move horizontally across species barriers ensuring their long term persistence within populations or among fungal mitochondrial genomes^28^.

The current study characterized the mitochondrial genomes of four strains of *E*. *resinifera*. The most noteworthy findings for these large (>214 kbp) genomes were the large numbers of introns and their IEPs. In addition, other components such as the protein-coding genes and other genetic components such as intergenic regions and remnants of inserted plasmids are also being described. These intron-rich genomes provide an opportunity to examine the mobility of group I introns and their HEGs. Currently, the actual mechanisms for intron acquisition and loss are still poorly understood. With regards to intron content one could speculate that ancestral mtDNAs were intron-rich and they are gradually being eroded and lost or alternatively, introns are continuously lost and reacquired by outcrossing or horizontal transfer^27,28^. The *E*. *resinifera* mitochondrial genomes were compared among the four strains examined, and also with three related mitochondrial genomes from *C*. *platani*, *C*. *cacaofunesta* and *C*. *fimbriata* in order to gain a better understanding of the evolutionary mechanisms that could promote intron loss or gain and mitochondrial genome rearrangements.

## Results

### Mitochondrial genome of *E*. *resinifera*

**Figure 1:**
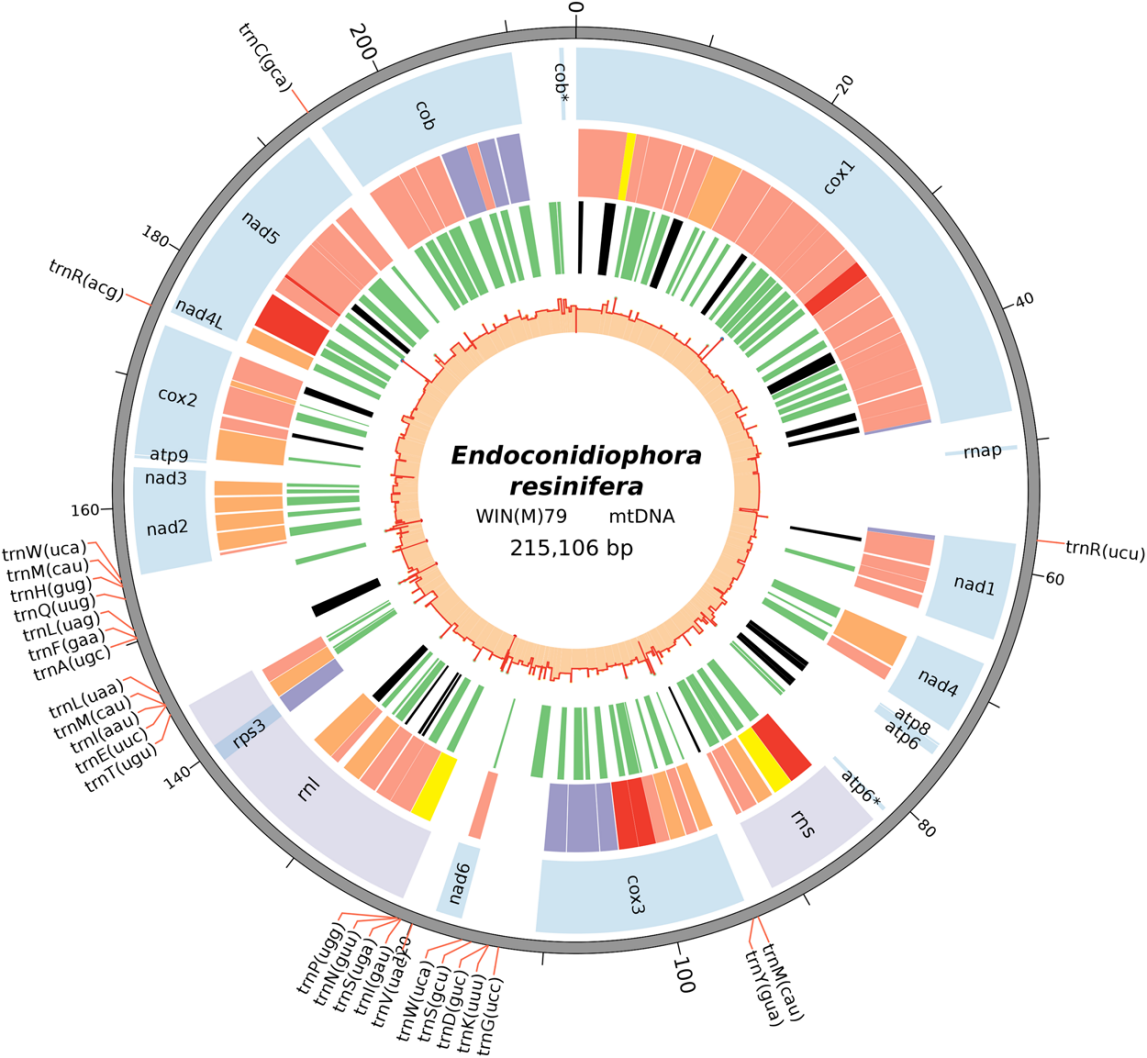
The annotated mitochondrial genome of *E*. *resinifera* [strain WIN(M)79]. The total size of this circular genome is 215 kb (represented by the scale). The position of the tRNAs are shown on the outer track, with the positions connecting to the scale with red lines. The first inner circle represents the position, size and the names of the protein-coding and rRNA genes. The introns are shown in the second inner circle and are colour coded according to the intron types/subtypes: group II (yellow), group IA (purple), group IB and group I derived (very light red), group IC (orange), and group ID (dark red). The third inner circle is to visualize the presence of the LAGLIDADG (green) or GIY-YIG (black) homing endonuclease genes encoded by the introns. The innermost circle is the GC plot of this genome; calculating GC% of genome features. Note the third inner circle also shows the location of free-standing homing endonuclease genes.

The mitochondrial genomes for four *E*. *resinifera* strains [WIN(M)79 (=UAMH 9644), WIN(M)1409 A, WIN(M)1410B, WIN(M)1411] were sequenced, assembled and annotated (GenBank accession numbers: MH551223, MK026450, MK026449, MK012641 respectively). The mitochondrial genome for strain WIN(M)79 was annotated first and this genome was used as the reference mtDNA for this study; overall, the four *E*. *resinifera* mtDNAs only differed by the absence or presence of 4 introns and 2 single nucleotide polymorphisms. The genome of *E*. *resinifera* is composed of protein-coding genes such as (*atp6*, *atp8*, *cob*, *cox1-3*, *nad1-6* and *nad4L*) and rRNA (*rns* and *rnl*) and tRNA (27 tRNAs) genes. Most of the protein and rRNA -coding genes were noted to be populated with group I and group II introns, and most introns encode open reading frames (iORFs). The mitochondrial genomes of the *E*. *resinifera* strains were annotated in Artemis and the genome for strain WIN(M)79 was visualized in Circos (Fig. 1).

### Protein-coding, rRNA and tRNA genes

The mitochondrial genome of *E*. *resinifera* contains 14 protein-coding genes and this includes NADH dehydrogenase subunits (*nad1*, *nad2*, *nad3*, *nad4*, *nad4L*, *nad5* and *nad6*) which contribute towards the electron transport complex I, cytochrome oxidase subunits (*cob*, *cox1*, *cox2* and *cox3*) that are part of complex III and complex IV, ATP synthase subunits (*atp6* and *atp8*) and the gene that encodes the 40 S ribosomal protein S3 (*rps3*). The genome contains the small and large ribosomal RNA genes (*rns* and *rnl*). A total of 27 tRNA genes were identified of which most are located around the *rnl* gene; 10 tRNA genes are upstream and 12 are downstream of the *rnl* gene although the upstream tRNA cluster is interrupted by the *nad6* gene. The remaining 5 tRNA genes are dispersed along the genome. All of the protein-coding genes, rRNA and tRNA genes reside on the same strand of the mtDNA. The gene sizes, positions and arrangements are depicted in Fig. 1.

### Introns and intron-encoded ORFs

Eighty-one introns were found within the mitochondrial genome of *E*. *resinifera* WIN(M)79 and 72 of them contain ORFs coding for homing endonucleases (HEs). Among the 81 introns, according to RNAweasel^29^, 12 can be assigned to group-IA, 32 to group-IB, 17 to group-IC, 8 to group-ID, 9 to group-I(derived) introns (Supplementary Table 1) and three introns have features diagnostic for group II introns^30–32^. Seventy-two group I introns in *E*. *resinifera* encode one or two iORFs. In total, 76 LAGLIDADG and 15 GIY-YIG type ORFs were identified within the *E*. *resinifera* group I introns. Group II introns can code for either homing endonuclease or reverse transcriptases^31^. In this study we observed three group II introns. Two of them (located in *rns* and *rnl* gene) encoded LAGLIDADG type ORFs. Another group II intron (located in *cox1* gene) appeared to have no ORF.

### A tandem intron (mS917a and b) in the *rns* gene

**Figure 2:**
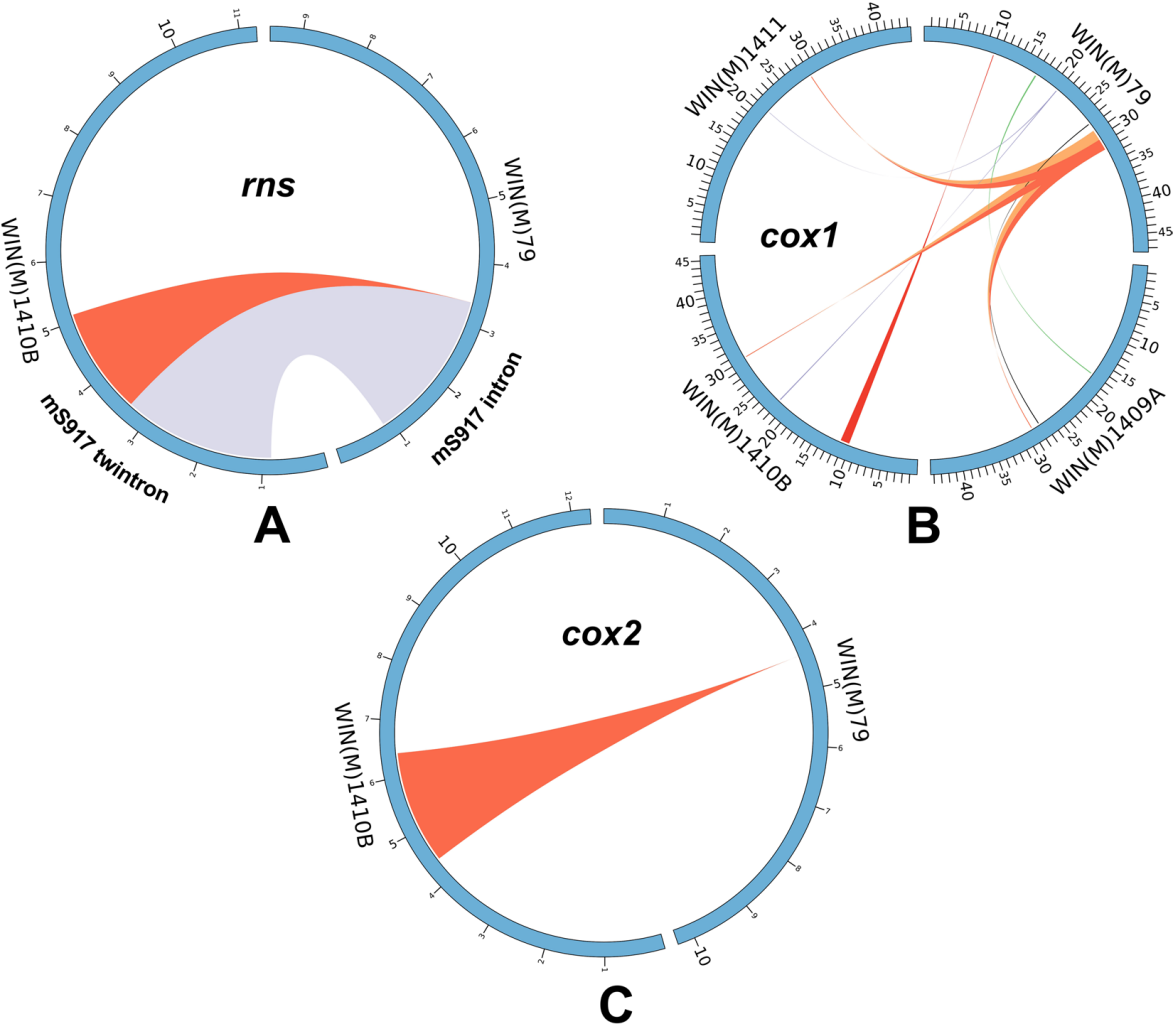
The comparison of *rns*, *cox1* and *cox2* genes from four strains [WIN(M)79, WIN(M)1409 A, WIN(M)1410B, WIN(M)1411] of *E*. *resinifera* considering the strain WIN(M)79 as a reference. (**A**) Comparing the *rns* gene showed that there is one novel group ID intron in WIN(M)1410B which is referred as a tandem intron (mS917). (**B**) Comparison of *cox1* genes showing that there are two additional introns in WIN(M)79 and one additional intron in WIN(M)1410B, moreover there are indels in different intronic regions. (**C**) With regards to the *cox2* gene WIN(M)1410B has one additional intron compared to the other strains.

Among the four strains of *E*. *resinifera*, the WIN1410B strain showed a unique type of intron arrangement within the *rns* gene (Fig. 2A). All strains analyzed for this species have a group ID intron at position S917 (intron insertion sites designated according to Johansen and Haugen^32^). The other available *Ceratocystis* species also contain the mS917 intron. However, *E*. *resinifera* WIN(M)1410B has two group ID introns (instead of one) side by side (a and b) without any apparent exon sequence separating them. This special arrangement could be termed “tandem intron” or a “side by side” twintron; for consistency we will refer to this arrangement as a tandem intron. The mS917a and mS917b intron-encoded ORFs of the tandem intron are both related to the mS917 clade of LAGLIDADG ORFs previously characterized^10,33^. Phylogenetic analysis showed that the components of the tandem intron mS917a and b ORFs are paralogues and other members of this clade can be located in group ID introns located within the *rnl*, *nad5*, *nad6* and *cox3* genes. Moreover, the intron ORF from the mS917b component groups with the intron ORFs located in the *rnl* intron (mL2029) clade, whereas the mS917a ORF groups with orthologues located within the *rns* mS917 intron. Based on the phylogenetic distribution of members of the 917 family of HEs it would appear that the mS917b intron/ORF is derived from a version of the mL2029 intron that has inserted (ectopically) immediately after the mS917a intron (Supplementary Fig. 1).

### GC percentage and composition of the genome

The mitochondrial genome of *E*. *resinifera* is AT-rich (71%). The average GC content of the genome is 29% which is maintained across the mtDNA. The tRNA genes, however, have a higher GC (up to 50%) content compared to the rest of the genome. The genome is composed of genes (exons and introns), rRNAs (exons and introns), tRNAs, intron-encoded ORFs, and intergenic regions. However, the majority of the mitochondrial genome is comprised of introns and intron-encoded ORFs (68% of the genome). The introns embedded within the protein-coding genes make up 56% of the entire genome. The second major component of these genomes is comprised of the introns present within the rRNA genes (12%). The nucleotide sequences for protein-coding sequences (CDS) and rRNAs comprise 7% and 3% of the mitochondrial genome, respectively. The tRNAs make up 1% of the genome, and 21% of the mitochondrial genome is comprised of intergenic sequences. With regards to the 68% intron component, it can be arranged as follows for the intron subtypes: group IA, IB, IC, ID, I (derived) and group II as follows 14%, 42%, 22%, 9%, 10% and 3% respectively. The iORFs occupy half (50%) of the intron bases. The LAGLIDADG ORFs comprise 42% and the GIY-YIG ORFs make up 8% of all the bases that make up the introns (Supplementary Fig. 2).

### The largest *cox1* gene recorded so far among the Ascomycota

The mitochondrial genome of *E*. *resinifera* is one of the largest [215 kb for WIN(M)79 and 220 kb for WIN(M)1410B] mitochondrial genomes so far reported for a member of the Ascomycota and it also contains the largest *cox1* gene (47.5 kb) recorded so far for any fungus. The size of this genome is for the most part due to the large number of introns (81 introns for WIN(M)79) and the *E*. *resinifera* mitochondrial genome in comparison to the other *Ceratocystis* spp. appears to have higher numbers of introns (Table 1). The *cox1* gene also appears to have expanded in *E*. *resinifera* to 47.5 kb due to the large number of introns (23 introns). Among the 23 introns, 22 are group I introns and one group II intron was identified. The group IB is the most abundant (17 group IB introns) intron type in the *cox1* gene (Supplementary Table 1). This gene appears to be rich in introns compared to other genes in the mitochondrial genome. The *cox1* gene is a very conserved gene (at the CDS level), but the intron numbers are variable among different strains examined for *E*. *resinifera* and also variable among species of *Ceratocystis* (Table 1). Among strains of *E*. *resinifera cox1* intron numbers are 21 for strains WIN(M)1409 A and 1411, 22 for strain WIN(M)1410B and 23 for strain WIN(M)79. This is in contrast to *C*. *platani*, *C*. *cacaofunesta* and *C*. *fimbriata* where the number of *cox1* introns is 9, 10 and 12, respectively.

**Table 1:** Comparison of the mitochondrial genomes of the *E*. *resinifera*, *C*. *cacaofunesta* (JX185564.1), *C*. *platani* (LBBL00000000.1) and *C*. *fimbriata* (APWK03000239.1).

Organism	mtDNA size (bps)	GC%	Number of introns (per gene)													Total introns
			*cox1*	*nad1*	*nad4*	*atp6*	*rns*	*cox3*	*nad6*	*rnl*	*nad2*	*cox2*	*nad4L*	*nad5*	*cob*	
*C*. *cacaofunesta*	103,147	26	10	1	1	2	1	2	1	3	0	6	1	7	2	37
*C*. *platani*	116,162	27	9	2	1	2	2	4	0	5	1	5	1	6	3	41
*C*. *fimbriata*	141,204	27	12	3	1	2	1	4	0	4	2	6	1	10	5	51
*E*. *resinifera* WIN(M)79^a^ (=UAMH 9644^b^)	215,106	29	23	5	2	0	5	9	1	11	5	5	1	7	7	81
WIN(M)1409 A	215,081	29	21	5	2	0	5	9	1	11	5	5	1	7	7	79
WIN(M)1410B	220,224	29	22	5	2	0	6c	9	1	11	5	5	1	7	7	81^c^
WIN(M)1411	214,750	29	21	5	2	0	5	9	1	11	5	5	1	7	7	79

^a^WIN(M) culture collection of J. Reid, Department of Microbiology, University of Manitoba, Winnipeg, Manitoba, Canada.

^b^UAMH Centre for Global Microfungal Biodiversity, Division of Occupational & Environmental Health, Dalla Lana School of Public Health, University of Toronto, Toronto Ontario, Canada.

^c^For WIM(M)1410B the tandem intron located in the *rns* gene (mS917) was counted as two introns.

### Open Reading Frames and gene fragments within the intergenic spacers: HEGs and a plasmid-derived RNA polymerase

Twenty ORFs were detected in the intergenic regions of the mitochondrial genome of *E*. *resinifera*. A blastp search of those ORFs against the NCBI non-redundant database showed the presence of a partial DNA-dependent RNA polymerase (rnap) gene which showed similarity with a mitochondrial plasmid encoded rnsp gene in *Neurospora intermedia*^34,35^. Previously, a degenerated RNA pol gene was also reported from *C*. *cacaofunesta*. Nine degenerated GIY-YIG and eight LAGLIDADG (degenerated) ORFs along with partial duplications of the *atp6* and *cob* genes were also recorded from the intergenic spacers (Supplementary Table 2; Fig. 1). The partial duplication (C-terminal segment) of the *cob* gene is located downstream of the *cob* gene (genomic position: 213807–214152). There are three degenerated LAGLIDADG ORFs situated in the intergenic space between *cob* and the partial C-terminal duplication of the *cob* gene. The *atp6* gene is followed by a C-terminal duplication of the *atp6* gene and this duplicated segment is located between the *atp6* and *rns* genes (genomic position: 81091–81417). The duplicated *atp6* segment is flanked by 5 GIY-YIG and 2 LAGLIDADG type ORFs. Partial C-terminal duplications of the *cob* and *atp6* genes were noted in all four strains of *E*. *resinifera*. In strain WIN(M)1411 the complete duplication of the *trnA* gene was recorded and the duplicated version also included some of the upstream bases associated with the original copy of *trnA* (genomic position 149128–149198 duplicated at 149958–150028).

### Degenerated *atp9*

The ATP synthase subunit 9 (*atp9*) gene sequence is present in the mitogenome of *E*. *resinifera* [genomic position: 163871–164096 in WIN(M)79] but it appears to have degenerated due to the presence of a premature stop codon. Blastx analysis showed a strong match (70% identity) to the *atp9* gene of *C*. *cacaofunesta* (GenBank accession YP_007507043.1). The same phenomenon was noted for *C*. *platani* (GenBank accession LBBL00000000.1) where its *atp9* gene sequence showed near 100% identity with the *atp9* sequence of *C*. *cacaofunesta*, but the *C*. *platani atp9* sequence was also interrupted by a premature stop codon. It is noteworthy that the *atp9* gene is absent in the *C*. *fimbriata* mitogenome (GenBank accession APWK03000239.1). Using the mitochondrial *atp9* amino acid sequence of *C*. *cacaofunesta* as a query against the *C*. *fimbriata* and *C*. *platani* genome data (including translated protein sequences) in a blastp analysis we noted that *atp9* sequences were located on the nuclear contig LBBL01000195.1 for *C*. *platani* and the nuclear scaffold APWK03000057.1 for *C*. *fimbriata*. The nuclear *atp9* gene products are also available from Genbank for *C*. *platani* (KKF93962.1) and *C*. *fimbriata* (PHH52759.1).

### Mitochondrial genome comparison

The genomes of *E*. *resinifera* strains are highly conserved with polymorphism mainly due to the presence or absence of introns along with some short insertions and nucleotide substitutions in the non-coding sequences. The differences among the strains in gene sequences (including intron and exon) are compiled in Supplementary Table 3. Briefly, it was found that the CDS of gene sequences were highly conserved among the strains; only one silent mutation was noted in the *cox1* gene in the WIN(M)1410B strain (Supplementary Fig. 3). The *rnl* gene of WIN(M)1409 A and WIN(M)1410B showed small insertions and the *trnA* gene of WIN(M)1411 showed a small insertion (see Supplementary Table 3). But sources of mitochondrial genome variability among the strains are due to variations of the number of introns along with some small indels in the intronic sequences. Additional introns were noted in the *rns*, *cox1* and *cox2* genes of strain WIN(M)1410B (Fig. 2A,B). Moreover, two additional introns were found in the *cox1* gene of strain WIN(M)79 (Fig. 2B). We found no significant variations with regards to intergenic regions.

For a more detailed comparison among *Ceratocystis sensu lato* species, we have collected the mitochondrial genome sequences for *C*. *cacaofunesta*, *C*. *platani* and *C*. *fimbriata*. *Ceratocystis cacaofunesta* is fully annotated (GenBank accession: JX185564.1), and *C*. *platani* is available as one contig (but was not annotated) in GenBank (LBBL00000000.1) and in ENA database (GCA_000978885.1). The mitochondrial genome for *C*. *fimbriata* is available in GenBank as a single contig (APWK03000239.1) but also not annotated. We have annotated *C*. *platani* and *C*. *fimbriata* for this study. The protein-coding regions were translated and extracted to compile a concatenated dataset that allowed these fungi to be compared with each other along with other members of the Ascomycota. The phylogenetic analysis showed that the *Ceratocystis s*. *l*. spp. are distinct from each other and they do comprise a separate clade (Microscales) in the phylogenetic tree based on concatenated mtDNA protein-coding sequences (Supplementary Fig. 1). All four members of *Ceratocystis s*.*l*. grouped into one clade with *E*. *resinifera* forming the basal member and sequences for *C*. *platani* and *C*. *fimbriata* grouping together. The phylogenetic tree overall showed strong node support values for the major nodes and the Microascales were positioned between the following Orders Hypocreales and Glomerellales (Supplementary Fig. 4).

**Figure 3:**
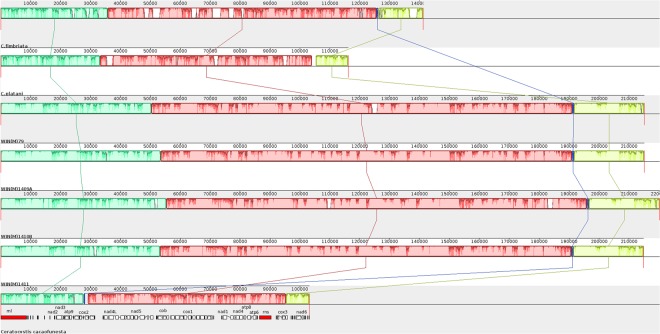
Genome-wide comparison for species of *Ceratocystis* Mauve. The progressiveMauve alignment (in Mauve program) shows the homologous blocks shared among the mitochondrial genomes and it also connected these blocks with lines, indicating corresponding position among the homologous blocks in order to visualize the gene arrangement.

The variation in genome sizes, GC content and the presence of introns in every gene among the *Ceratocystis s*.*l*. species are listed in Table 1. The data showed that the genome size and intron number of *E*. *resinifera* is about double compared to other *Ceratocystis* species. A comparative alignment of all these mitochondrial genomes was done with the Mauve program (Fig. 3). It clearly showed the homologous blocks shared between these genomes and it also showed a linear relationship among the genes, which implies that the gene order or synteny of these genomes is conserved. Besides the gene synteny for protein and rRNA coding genes, the comparison of the tRNA genes also showed conservation of gene order. However, the number of tRNA genes is not the same among the examined species: 25 tRNAs in *C*. *fimbriata*, 26 in *C*. *platani*, 27 in *E*. *resinifera* and 30 in *C*. *cacaofunesta* (Supplementary Table 4).

## Discussion

### Mitochondrial genome architecture among members of *Ceratocystis sensu lato*

The phylogenetic tree generated for ascomycetes fungi, based on concatenated mitochondrial protein sequences, generated a well-supported topology consistent with the topologies of previously published reports based on rDNA data^2,36^. *Ceratocystis* and allied taxa belong to the Microascales and are distinct from species that can be assigned to other orders such as the Hypocreales, Glomerellales, Xylariales, Sordariales, Ophiostomatales etc. The assembly of the mitogenomes for the tested strains of *E*. *resinifera* can be represented as circular molecules ranging in size from 214,750 to 220,224 nucleotides. It should be noted that fungal mtDNAs could also have linear topologies and have been proposed to occur as long concatemers, possibly products of a recombination-dependent rolling circle-type DNA replication mechanism^37–39^.

The mitochondrial genomes of *E*. *resinifera* are the largest genomes reported so far for members of the Ascomycota; yet with regards to the standard mtDNA core genes these genomes do not offer additional genes compared to other fungal mitochondrial genomes^40–42^. The *E*. *resinifera* mitochondrial genome contains 15 protein coding genes, 2 rRNA genes and 27 tRNA genes similar to other fungal mitochondrial genomes. Moreover, the *rps3* gene is embedded within an *rnl* group IA type intron (mL2449), which is a common feature in many filamentous ascomycetes fungi mitogenomes^43^. The *atp9* gene apparently is found to be present in some fungi and but not in others^44,45^. With regards to the mitochondrial genomes examined in this study the *atp9* gene is present in *C*. *cacaofunesta*, but in *E*. *resinifera* and *C*. *platani* the *atp9* gene has accumulated mutations that generated a premature stop codon and in *C*. *fimbriata* the *atp9* gene is missing. This would suggest that the *atp9* gene is drifting in some species and a nuclear counterpart might be available that can compensate for the loss of the mitochondrial version of the *atp9* gene. Indeed examining the nuclear genomes of *C*. *platani* and *C*. *fimbriata* uncovered nuclear encoded versions of *atp9* suggesting that a copy of the mtDNA *atp9* gene has been transferred to the nuclear genome in these fungi. Similar findings were noted for *Stemphylium lycopersici* where *atp9* is missing from the mitochondrial genome but a complete version could be located in the nuclear genome^44^.

The progressiveMauve alignment of the mitochondrial genomes for the *Ceratocystis s*.*l*. species showed that gene synteny is conserved and variations in mtDNA and gene sizes are mostly due to the expanding numbers of introns. Variation among various strains of *E*. *resinifera* is restricted to one synonymous substitution in the *cox1* gene, a few SNPs within the intronic sequences or other non-CDS bases along with a few indels within the intergenic regions. Similar to what has been observed in other fungi such as *Chrysoporthe* species^46^, *Aspergillus* and *Penicillium* species^21^, and *Saccharomyces sensu stricto* species^47^, we observed intron derived size polymorphism among strains of *E*. *resinifera*.

Other noteworthy features are the fusions of several gene pairs typically involving a one nucleotide overlap among the two reading frames. The overlap of *nad2* with *nad3* genes and *nad4L* with *nad5* by one nucleotide has been noted in other fungi^22^.

### Mobile elements and genome expansion (duplication and degeneration)

Recent papers have noted that fungal mitochondrial genomes are dynamic with regards to their structure and composition due to the presence of mobile elements (such as group I and group II introns) and duplication events^20,42,48^. This study found 81 introns in the mtDNAs of *E*. *resinifera* strains examined which is nearly double the number of introns compared to the other species of *Ceratocystis*. Most noteworthy is the *cox1* gene from *E*. *resinifera* strain WIN(M)79 that has 23 introns and this gene is 45.7 kb long. The size of the *cox1* gene alone exceeds the sizes of many complete fungal mitochondrial genomes^49^. The *E*. *resinifera cox1* gene is the largest reported so far, previously the *cox1* gene from *Agaricus bisporus* at ~30 kb long with 19 introns was reported to be the largest *cox1* gene among the fungi^50^. In *E*. *resinifera* the *cox1* gene is considerable longer and acquired 4 more introns; combined with the intron encoded ORFs this expanded the size of the gene to 45.7 kb. The *cox1* gene has been utilized as a DNA barcoding marker in metazoans, but the presence of potentially large numbers of introns makes the *cox1* gene not very suitable for fungal DNA- based barcoding^51^. Mobile elements that require specific target sequences such as group I and group II introns favor genes that are under functional constraints and are highly conserved, making intra- and intergenomic mobility more feasible.

Examples of degenerated intron ORFs were noted and these are to be expected as according to Goddard and Burt^27^ introns and encoded ORFs such as homing endonucleases are not subject to natural selection, thus their sequences drift and can accumulate deleterious mutations. Neutral evolution is thus a plausible model to explain the potential genome expansion noted among some members of *Ceratocystis s*.*l*. Although introns appear to be the major factor that contributes towards mtDNA size expansion in *E*. *resinifera*, insertion of plasmid components (such as the rnsp gene), and gene duplication events (partial duplication of *atp6*, *cob* and HEGs) and the expansion of intergenic spacers also contribute towards the size of the mitochondrial genome.

Overall, the examined mitochondrial genomes for *Ceratocystis* and *Endoconidiophora* species appear to evolve rapidly in gene structure (i.e. intron composition) but slowly in sequence and gene order. This has also been observed in plant mitochondrial genomes and some fungi^46,52^. Therefore, our findings show that mtDNA polymorphisms are mostly due to the presence and absence of introns.

### Tandem intron located at mS917

So called twintrons have been described from various fungal mitochondrial genomes with various combinations of group I or group II introns nested inside each other. These elements may require that during RNA processing the internal member has to splice first before the external member can be excised from the transcript^53^. Deng *et al.*^49^ noted that in *Hypomyces aurantius* the *cox3* gene harbored a twintron (cox3-i2) that is a “side-by-side twintron” where two group IA introns are arranged in tandem. The *rns* gene in *E*. *resinifera* [WIN(M)1410B] contains a twintron where two group ID introns are placed next to each other at the S917 position of the *rns* gene. This position (S917) has previously been noted to be invaded in some fungi by a group ID intron that expresses active HEases^34^, in addition it has been recorded that this location in *Cryphonectria parasitica* can be occupied by a twintron where a group ID intron that encodes a double motif LAGLIDADG-type ORF is inserted into an ORF-less external group ID intron^11,54^. This arrangement differs from that observed in *E*. *resinifera* (strain WIN(M)1410B) where two LAGLIDADG ORF-encoding group ID introns are situated next to each other. Based on the phylogenetic relationships between the two members of this tandem intron, it appears the 5′ member is the original resident of the S917 site and the 3′ component is due to an ectopic integration event whereby a paralog of the mS917 HEG which was probably located in the *rnl* gene reinvaded the mS917 position. This intron arrangement warrants further characterization in future studies with regards to its splicing pattern and the target preferences for the intron encoded HEases.

### Evolutionary dynamics of the introns and HEGs and the mitochondrial genome

Introns comprise 68% of the mitochondrial genome in *E*. *resinifera*, and most of the introns contain ORFs encoding putative homing endonucleases. Those ORFs comprise 50% of the size of the introns. Group I introns can move to cognate alleles that lack introns or in some instances, ectopically integrate into new sites, as they encode homing endonucleases^25^. Intron-loss can be mediated when a reverse-transcribed mature transcript replaces the original intron-containing gene^55^. Deletion of introns can also be due to intra- or intergenomic recombination events^56^. The evolutionary dynamics of introns and homing endonucleases is quite complex, the gain and loss of introns and their encoded ORFs tends to be attributed to a HEG lifecycle^27^ that is based on neutral evolution. The model is based on the observation that among members of the Saccharomycetales the omega intron (*rnl* gene introns) appears to undergo a cycle of invasion and degeneration; as there is no selection, introns and encoded ORFs accumulate mutations that eventually lead to their erosion and loss. To persist these elements have to keep invading cognate intron-minus alleles, transpose into new sites or move horizontally into new genomes, or gain a beneficial function^27,28^.

The large number of introns noted in some fungal mitochondrial genomes such as *Agaricus bisporus*, *Rhizoctonia solani*, *Cryphonectria parasitica*, *Sclerotinia borealis*, *E*. *resinifera* etc. are in contrast to small fungal mtDNAs encoding only one intron such as *Sporothrix schenckii* and *S*. *globosa*; this raises the question if drift is indeed the only possible explanation for the distribution of introns within fungal mitochondrial genomes? Also, some introns appear to be quite conserved such as the *rnl* intron that in many ascomycetes fungi encodes *rps3*. It is assumed that encoding a potentially essential gene ensures these introns are not subject to neutral evolution. Other introns for less obvious reason also appear to be observed at relative higher frequencies compared to other introns, such as cob393 and cob490 and possibly these introns may provide some means for gene regulation and therefore selection may favour their maintenance^57^. Rudan *et al.*^58^ recently presented data from *S*. *cerevisiae* that suggests the mtDNA introns are important in fine-tuning gene expression and they facilitate the generation of appropriate amounts of transcripts. Belfort^59^ has suggested that some self-splicing elements could be bio-sensors that can modulate the expression of the genes that contain introns or inteins. Conversely, many introns (intron insertion sites) have a rather disjointed distribution among the fungi encoding ORFs at various state of degeneration; these introns may be excellent examples of neutral evolution as proposed by Goddard and Burt^27^.

**Figure 4:**
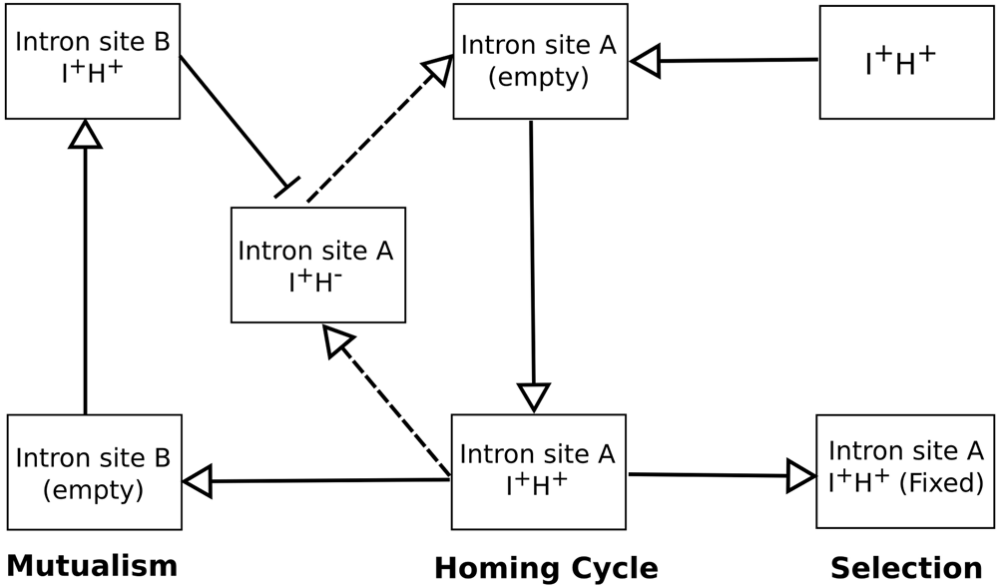
The fate of composite elements such as introns plus IEPs (I + H+). The composite element invades an empty site and from here it could spread into other sites (only site B shown for simplicity) and related IEPs could still interact with their ancestral intron version possibly facilitating splicing or mobility thus enhancing the chances of the ancestral intron to persist. This form of mutualism could even complement situations where the ancestral intron ORF has started to accumulate deleterious mutations (H−). Other composite elements may be strictly subject to drift and first the ORF is degenerating and eventually the intron is lost from the genome and possibly from the population. There might be situations where the composite elements have been co-opted as maturases or regulatory elements or as platforms for expressing essential genes (*rps3*) and these introns would be subject to adaptive selection and thus could become fixed in the genome and the population (Figure adapted from Gogarten and Hilario^83^; licensed under a CCBY 2.0 license, (https://creativecommons.org/licenses/by/2.0/). This Figure adds a new component to the standard homing endonucleases life cycle^27,83^ that suggests that some HEGs can avoid extinction due to mutualistic interactions that can complement for the accumulation of mutations.

Another category of introns is represented by those that appear to have invaded new sites within the same genome^60^, a temporary means of escaping the Goddard and Burt^27^ HEG lifecycle of invasion, decay and eventual loss. Related introns present within the same genome may still interact in *trans* in some collaborative fashion rendering them less prone to extinction^61,62^. Some intron encoded proteins (IEPs) can also act as maturases that facilitate the intron RNA to fold into a splicing-competent configuration^29^. In *S*. *cerevisiae*, two homologous IEPs have been characterized, and cytb bI4 is required for splicing of both the cytb bI4 intron and the cox1 aI4 intron but the cox1 aI4 IEP can generate double stranded cuts within *cox1* sequences^63^. Trans-acting interactions between introns and free standing HEGs have been noted among phages; collaborative homing refers to scenarios where a HE can catalyze the mobility of an intron as both share the same insertion/target site^64–66^. In scenarios such as the mS917 clade of introns where orthologous elements have spread into the *rns*, *rnl*, *nad5*, *nad6* and *cox3* genes^34^ one can propose that there might be some interactions among members of this clade. *E*. *resinifera* has mS917 members located within the *rns*, *rnl*, *nad5* and *nad6* genes in addition one strain (WIN(M)1410B) contains a tandem intron located at mS917 composed of two members (*rns* and *rnl* version, Fig. 2A) of this family of introns. When members of a HEG family are present within the same genome one can envision a “hypercycle”-like analogy^67,68^; however, here dependencies are more lose as individual members can drift, become selfish and some members can be short circuited^69^. Members would not have to interact in a directional manner instead interactions such as trans-acting maturase activities or trans-acting homing activity mostly likely would be linked to those that recently diverged from each other. This arrangement would provide some degree of stability for the persistence of members of a HEG family allowing members to maintain their numbers or even spread to new locations “outpacing” drift as predicted by the Goddard and Burt model^27^. These types of interactions (Fig. 4) may in part explain why some mitochondrial genomes have expanded by gaining or maintaining large numbers of introns.

Horizontal gene transfer, inter and intra-genome intron mobility, plus gene conversion promoted by IEPs and host genome repair systems combined with drift are the mechanisms that appear to promote intron diversity and potentially intron expansion in fungi^20,60,69,70^. Why are some fungal genomes almost completely devoid of introns? This could be again due to drift and the biased transmission of mtDNAs that are reduced in size or replicative advantage of smaller mtDNAs or loss of mtDNA introns could be the result of nuclear genome rearrangements that result in the loss of factors that can promote intron splicing, intron RNA stability, intron homing or mtDNA DNA repair (recombination).

## Conclusion

So far, few mitochondrial genomes are currently available for members of the Microascales. This study examines the mitochondrial genome of *E*. *resinifera* a species that used to be assigned to the genus *Ceratocystis*. The latter is a genus that has recently undergone extensive taxonomic revisions^71^ and the mitochondrial genome might offer mtDNA loci that could be developed into molecular markers assisting in the identification of taxa within this group of economically important fungi. Large mitochondrial genomes offer some insight on mechanisms that might cause these genomes to expand. With regards to *E*. *resinifera*, introns appear to be a major contributor towards genome expansion. Introns and their encoded homing endonucleases all assemble and initiate further invasions by drift^57^ but once they have inserted into a gene several mechanisms may determine their fate. Many probably do fit the model proposed by Goddard and Burt^27^ whereby these elements drift and thus face eventual elimination due to the accumulation of mutations and persistence within a population requires re-invasion of new loci or loci that lost introns. However, there appears to be evidence that some introns may actually be beneficial either encoding proteins that are useful to the genome (maturase activity, rps3) or introns that can act as gene regulators and thus these introns are maintained within a population. Finally, some introns appear to persist as they are co-operating with other introns promoting a system of mutualism that renders them less prone to extinction.

## Material and Methods

### Culturing fungi

The fungi were grown at 25 °C for 8–10 days on malt extract agar (MEA - 3% malt extract, 2% agar and 0.1% yeast extract) plates. Mycelium was scraped from these plates and transferred to 1 L flasks containing 250 mL yeast extract, peptone, dextrose broth (YPD - 0.1% yeast extract, 0.1% peptone, 0.3% dextrose). The YPD broth cultures were maintained for 8–10 days at 25 °C. The fungal strains of *E*. *resinifera* utilized in the study are listed in Table 1.

### Isolation of Mitochondria

Fungal mycelia was collected by vacuum filtration using a Büchner funnel and Whatman® qualitative filter paper. The mycelium was disrupted by grinding with mortar and pestle with the addition of 2 mL of isolation buffer [10 mM Tris-HCl (pH 8.0), 440 mM sucrose, 5 mM ethylene-diamine-tetra-acetic acid (EDTA)] and 1.5 g of acid-washed sand for each 1 g of mycelia. The fungal material was ground for about 5–10 minutes until the mycelia/sand/buffer mixtures forms a slurry. This slurry was transferred to a 25 mL Corex® centrifuge tube (ThermoFisher) and centrifuged for 15 min at 3000 g using a Sorvall® SS-34 fixed angle rotor in a Sorvall® RC-5B Plus centrifuge at 4 °C to pellet nuclei, cell debris and sand. The supernatant was transferred to a 25 mL Corex® centrifuge tube (ThermoFisher) and centrifuged at 20 000 g using a Sorvall® SS-34 fixed angle rotor in a Sorvall® RC-5B Plus centrifuge at 4 °C for 30 min to pellet the mitochondria.

### Mitochondrial DNA extraction

The mitochondrial enriched pellet was suspended in 3 mL of extraction buffer [100 mM Tris-HCl (pH 8.0), 2% cetyl-trimethylammonium bromide (CTAB), 20 mM EDTA, 1.4 M NaCl] plus 330 µL of 20% sodium dodecyl sulfate (SDS) and nucleic acids were extracted based on Hausner *et al*.^72^. Briefly the mixture was incubated for 2 hours (or overnight) at 55–65 °C and proteins and lipids were removed by adding an equal volume (~3 mL) of chloroform, after mixing the contents of the tube it was centrifuged at 3000 g with a IEC Centra CL2 centrifuge for 5 min. The top aqueous layer was transferred to a new 15 mL centrifuge tube and mixed with 4 µL of RNase A (QIAGEN) and incubated in a 55–65 °C water bath for 1 h to remove RNA. RNase was removed by addition of Chloroform in a 1:1 ratio in the tube and centrifuged at 3000 g for 20 min in an IEC Centra CL2 centrifuge. The aqueous layer was transferred to a new tube and mixed with 2.5 volumes of 95% ethanol and placed in the freezer at -20 °C for 1 hour. The mixture was then centrifuged at 3000 g for 15 min in an IEC Centra CL2 centrifuge to pellet the DNA. Supernatant was removed and the DNA pellet was washed with 1 mL of 70% ethanol, the tube was centrifuged again at 3000 g for 5 min and the ethanol was removed. The DNA pellet was air dried and suspended in 200 µL DNase/RNase-free water and placed in -20 °C for storage.

### Quantifying DNA

The extracted DNA was quantified with a NanoDrop 2000c UV-Vis Spectrophotometer and the quality was determined on the basis of the 260/280 and 260/230 OD ratio. Quantification was confirmed by gel electrophoresis of 10 µL of the extracted DNA sample on a 1% agarose gel.

### Genome sequencing and assembly

The mitochondrial genomic DNA was sequenced and assembled by Génome Québec (Innovation Centre, McGill University). For each sample 75 μL of DNA (~ 1 μg) supplied within an Eppendorf 96-well twin.tec® PCR plate (Cat. No. 951020401) sealed with VWR® aluminum foil (Cat. No. 60941–074), was sent to Génome Québec for Illumina sequencing using the MiSeq platform. The DNAs from different fungal samples were barcoded and combined into a single MiSeq run. The quality of the sequence reads were verified by FastQC^73^. The sequenced reads generated from NGS sequencing were assembled by the a5-miseq-pipeline^74^ – a MiSeq optimization of the original a5 pipeline^75^.

### Genome Annotation

The assemblage of the genome by a5-miseq pipeline yielded a set of scaffolds. The scaffolds were sorted out on the basis of the scaffold-size and presence of mitochondrial genes and those scaffolds were used to join and construct an entire uninterrupted mitochondrial genome sequence by using custom python script (available upon request) and NCBI-blast + program as well as by manual inspection. The position of the protein-coding genes, rRNAs and tRNAs were identified by MFannot^76^. tRNA genes were identified with tRNAscan-SE^77^. Intron-exon junctions within protein-coding and rRNA genes were initially obtained in MFannot and verified by multiple sequence alignments (MSA) of a gene and aligning it to the CDS of the same gene from related species. Sequence alignments were performed with MAFFT^78^. Sequences were also analyzed with the RNAweasel program^29^ to determine intron types and subtypes. Intron sequences were also examined with the ORF-Finder program (NCBI) to identify possible ORFs. Further, the Smart-BLAST program was used to determine the type of the intron-encoded ORFs. The coordinates of all genes, rRNA, tRNA, introns and intron-encoded ORFs and any other features were annotated using Artemis^79^ and visualized in Circos^80^.

### Genome comparison

The annotated *E*. *resinifera* mitochondrial genomes were compared for their features by generating multiple sequence alignments (MSA). The MSA allowed for noting SNPs, indels and polymorphisms that relate to the presence and absence of introns. For comparative purposes the mitochondrial genomes of *C*. *cacaofunesta*, *C*. *platani* and *C*. *fimbriata* were also included in the MSA.

A phylogenetic tree, based on a concatenated data set of 13 protein sequences (in alphabetical order: *atp6*, *atp8*, *cob*, *cox1-3*, *nad1-6*) was constructed in MrBayes^81^ based on an alignment of 41 fungal species generated with MAFFT. The tree topology generated by MrBayes was compared with previously published phylogenetic trees based on rDNA data^2,36^ and they were found to be consistent with one another. Also, a comparative map was generated in Mauve^82^ to visualize variations in the genomic architecture.

## Electronic supplementary material

Supplementary tables and figures

## Data Availability

All data nucleotide sequences generated are available in GenBank also data generated and analysed during this study are included in this published article (and its Supplementary Information files).
