# Supplementary material for: The mitochondrial genome of Endoconidiophora resinifera is intron rich
Source: Sci Rep. 2018 Dec 4;8:17591. doi: 10.1038/s41598-018-35926-y (PMC6279837; doi:10.1038/s41598-018-35926-y)
Supplement: Supplementary file 1 — Supplementary tables and figures [file 41598_2018_35926_MOESM1_ESM.pdf]

Supplementary Materials:

The mitochondrial genome of *Endoconidiophora resinifera* is intron rich.

Abdullah Zubaer, Alvan Wai, Georg Hausner*

Department of Microbiology, University of Manitoba, Winnipeg, MB R3T 2N2

*Corresponding Author

hausnerg@cc.umanitoba.ca

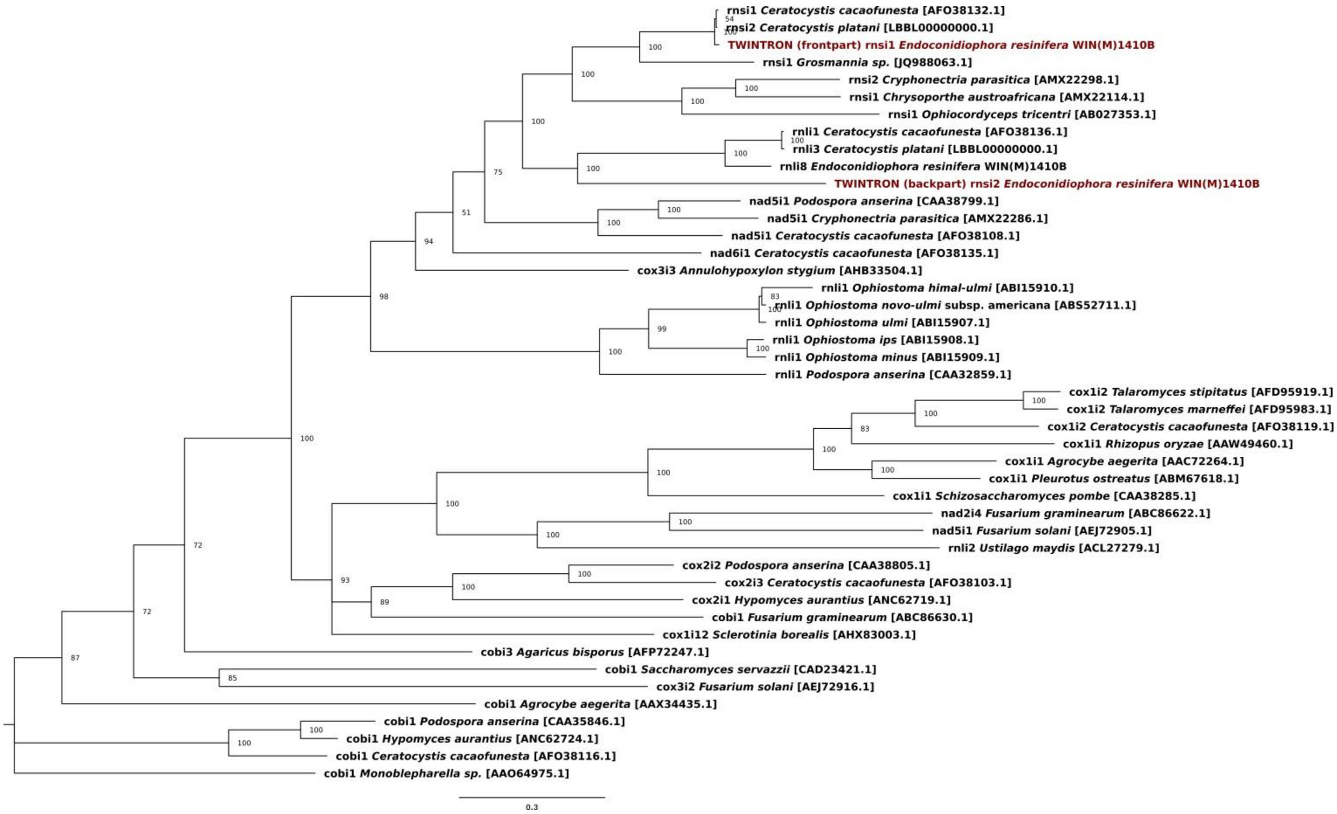

Supplementary Figure 1: Phylogenetic tree of the mS917 group ID introns encoded ORFs (from *rns* gene of *Endoconidiophora resinifera* WIN(M)1410B) and its homologues from different fungal species. The dataset included the amino acid sequences of 45 intron-encoded ORFs and processed for 1 million generation in MrBayes with the mixed-model default setting. Gene names (with its intron number), species name and NCBI accession number are mentioned for each species. The front part of the tandem intron (mS917) is found in the *rns* intron clade whereas the downstream component is clustered with the *rnl* intron ORFs.

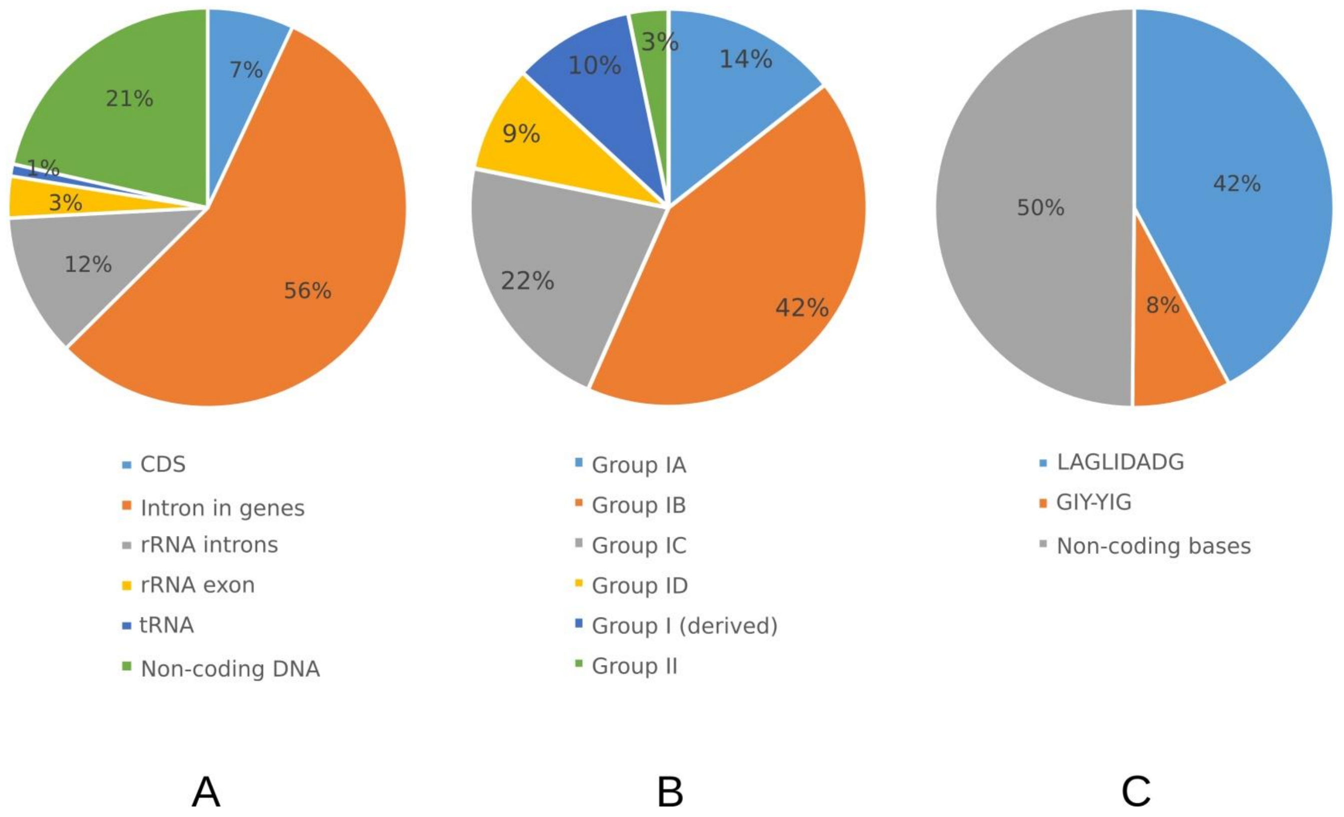

Supplementary Figure 2: Composition of the mitochondrial genome of *Endoconidiophora resinifera*. (A) The proportion of the protein coding genes (CDS and introns), rRNA genes (exon and intron), tRNA genes and intergenic (non-coding) sequences. (B) Introns (in protein coding and rRNA genes) are further categorised into their subtypes (such as group IA, IB, IC, ID, derived I, and group II). (C) Composition of the introns in terms of their encoded ORFs (either LAGLIDADG or GIY-YIG homing endonuclease gene) and non-coding bases in introns.

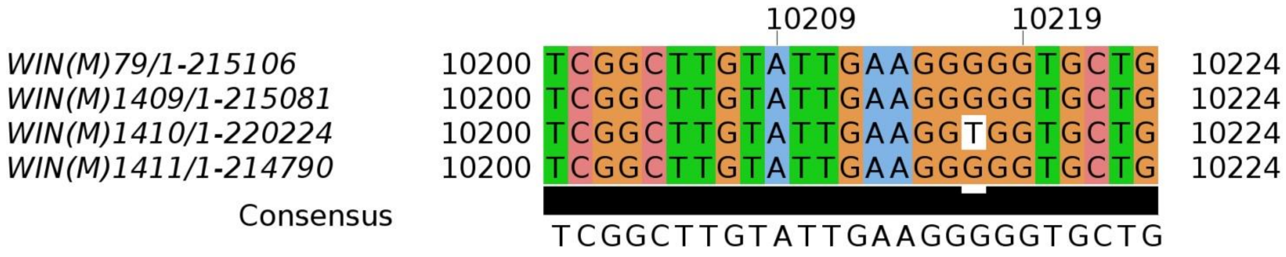

Supplementary Figure 3: Multiple sequence alignment of the four strains of *Endoconidiophora resinifera*. The represented region is a part of a CDS in *cox1* gene where the WIN(M)1410B strain has a single nucleotide polymorphism (SNP), a transversion of G to T. That is the only SNP found in the CDS region among those strains.

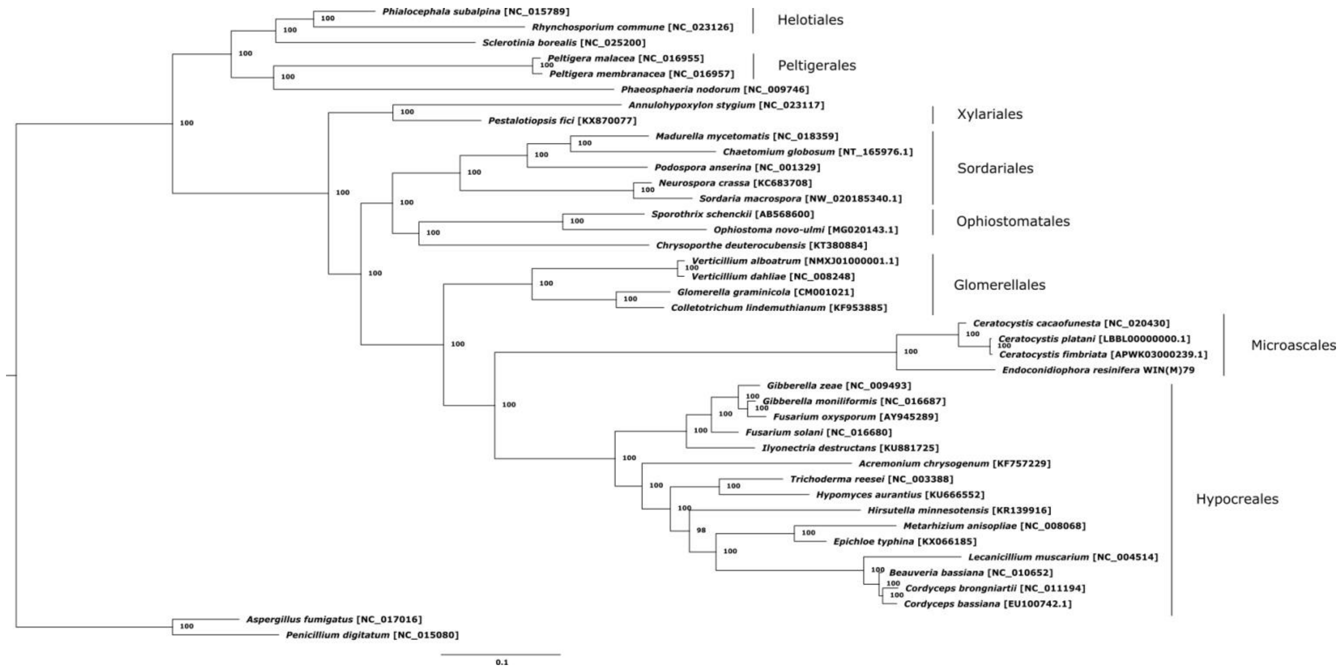

Supplementary Figure 4: Phylogenetic position of *Endoconidiophora resinifera* among the Ascomycota. The tree was inferred by Bayesian method in MrBayes using 1 million generation with the gamma-distributed rates model. The dataset was prepared by concatenating amino-acid sequences of the mitochondrial proteins extracted from NCBI genome database. The fungal species are clustered according to their orders in the tree rooted with *Aspergillus* and *Penicillium*. The *Endoconidiophora resinifera* placed with along with the related *Ceratocystis* species into a distinct clade.

Supplementary Table 1: List of introns (group I and group II) in each gene in *E. resinifera* strain WIN(M)79.

**Name of the gene**	**Group IA introns**	**Group IB introns**	**Group IC introns**	**Group ID introns**	**Group I (derived) introns**	**Total Group I introns**	**Group II introns**	**Total number of Introns**
cox1	1	17	1	1	2	22	1	**23**
nad1	2	3	-	-	-	5	-	**5**
nad4	-	1	1	-	-	2	-	**2**
atp8	-	-	-	-	-	-	-	**-**
atp6	-	-	-	-	-	-	-	**-**
rns	-	-	1	1	2	4	1	**5**
cox3	3	2	2	2	-	9	-	**9**
nad6	-	-	-	1	-	1	-	**1**
rnl	3	1	4	1	1	9	1	**11**
rps3	-	-	-	-	-	-	-	**-**
nad2	-	-	4	-	1	5	-	**5**
nad3	-	-	-	-	-	-	-	**-**
atp9	-	-	-	-	-	-	-	**-**
cox2	-	2	2	-	1	5	-	**5**
nad4L	-	-	1	-	-	1	-	**1**
nad5	-	3	-	2	2	7	-	**7**
cob	3	3	1	-	-	7	-	**7**
**Total**	**12**	**32**	**17**	**8**	**9**	**77**	**3**	**81**

Supplementary Table 2: List of the genetic components found in intergenic regions. Most of them are degenerated HEGs, however gene duplications and a RNA polymerase gene fragment were also found.

Gene name	Start (genomic position)	End (genomic position)
RNA Polymerase (rnap)	50255	50575
HEG (GIY-YIG)	75590	76156
HEG (GIY-YIG)	76179	76667
HEG (GIY-YIG)	76570	76890
HEG (GIY-YIG)	78102	78674
HEG (GIY-YIG)	78675	79124
HEG (LAGLIDADG)	79896	80213
HEG (LAGLIDADG)	80323	80727
ATP Synthase su.6 (atp6)	81091	81417
HEG (GIY-YIG)	92287	92598
HEG (LAGLIDADG)	111547	112203
HEG (LAGLIDADG)	112002	112985
HEG (GIY-YIG)	145826	146419
HEG (GIY-YIG)	146368	146736
HEG (GIY-YIG)	146670	147176
HEG (LAGLIDADG)	152425	153078
HEG (LAGLIDADG)	211869	212420
HEG (LAGLIDADG)	212342	212764
HEG (LAGLIDADG)	212868	213275
Cytochrome b (cob)	213807	214152

Supplementary Table 3: Notable differences in genes (exons and introns) sequences from different *E. resinifera* strains.

**Exon sequence in Gene (name)**	**Nucleotide position in the genome**				
	**WIN(M)79 (reference)**	**WIN(M)1409A**	**WIN(M)1410B**	**WIN(M)1411**	**Remarks**
rnl	131931	128784-130899	132034-134149		Insertion
	132449	131416-135650			Insertion (intron exon overlap)
cox1	10217		10217		Substitution (G to T)
trnA	150313			149242-150088	Insertion
**Intron sequence in Gene (name)**	**Nucleotide position in the genome**				
	**WIN(M)79 (reference)**	**WIN(M)1409A**	**WIN(M)1410B**	**WIN(M)1411**	**Remarks**
cox1	10226		10226-11548		Novel intron
	17008	17008-17041			Insertion
	20796		22117-22146	20796-20825	Insertion
	27592	27653			Deletion
	28800-30348	28859	30149	28828	Novel intron in WIN(M)79
	30360-32013	28869	30159	28838	Novel intron in WIN(M)79
rns	85745		83902-85657		mS917 Twintron
rnl	131931			128745-130860	Insertion
	132443-132457		134660-135555		Insertion
	132597		135695-135737		Insertion
cox2	168549		171691-173721		Novel intron
cob	203413-203430		208583		Deletion
	204218-204220		209371		Deletion
nad1	62101			58934	Substitution (A to T)

Supplementary Table 4: The order of the tRNA genes (trn*) from four different species. [The asterisk (*) represent an amino acid that is the product of the trn* gene]

**Organism**	**tRNA gene (trn*) order**																													
E. resinifera	R		M	Y	G	K	D	S	W	V		I	S	N	P	T	E	I	M	L	A	F	L		Q	H	M		W	R	C
C. cacaofunesta	R	I	M	Y	G	K	D	S	W	V	W	I	S	N	P	T	E	L	M	L	A	F	L	L	Q	H	M	M		R	C
C. platani	R		M	Y	G	K	D	S	W	V	W	I	S	N	P	T	E	L	M	L	A	F	L	L	Q	H	M				
C. fimbriata	R	I	M	Y	G	K	D	S		V	W			N	P	T	E	L	M	L	A	F	L	L		H	M	M			C
